# Identification of human thioredoxin as a novel IFN-gamma-induced factor: Mechanism of induction and its role in cytokine production

**DOI:** 10.1186/1471-2172-9-64

**Published:** 2008-11-05

**Authors:** Seol-Hee Kim, Jiyoung Oh, Ja-Young Choi, Ji-Young Jang, Myoung-Wha Kang, Choong-Eun Lee

**Affiliations:** 1 Laboratory of Immunology, Department of Biological Science, Sungkyunkwan University, Suwon 440-746 Korea

## Abstract

**Background:**

IFN-γ is a multifunctional peptide with a potent immune defense function which is also known as a prototypic Th1 cytokine. While screening for genes differentially expressed by Th1 and Th2 cytokines, human thioredoxin was identified as a novel target gene induced by IFN-γ. The mechanism by which thioredoxin is induced by IFN-γ and the signaling pathways involved in its induction were analyzed. In addition, the effects of thioredoxin on immune cell survival and cytokine production were examined by thioredoxin over-expression and recombinant thioredoxin treatment.

**Results:**

Human thioredoxin was selectively induced by IFN-γ in monocytic and T cell lines. In monocytic cells, the induction of thioredoxin gene expression by IFN-γ was dose-dependent, and both the mRNA and protein levels were increased by 2~3 fold within 4 to 24 h hours of IFN-γ treatment. The thioredoxin induction by IFN-γ was insensitive to cycloheximide treatment, suggesting that it is a primary response gene induced by IFN-γ. Subsequent analysis of the signaling pathways indicated that the Jak/Stat, Akt, and Erk pathways play a role in IFN-γ signaling that leads to thioredoxin gene expression. Thioredoxin was induced by oxidative or radiation stresses, and it protected the immune cells from apoptosis by reducing the levels of reactive oxygen species. Furthermore, thioredoxin modulated the oxidant-induced cytokine balance toward Th1 by counter-regulating the production of IL-4 and IFN-γ in T cells.

**Conclusion:**

These data suggest that thioredoxin is an IFN-γ-induced factor that may play a role in developing Th1 immunity and in the maintenance of immune homeostasis upon infection, radiation, and oxidative stress.

## Background

IFN-γ is a pleiotropic cytokine with a broad range of antiviral and immuno-modulatory actions. It is induced by various immune triggers and plays a critical role in directing cellular immune responses and inflammation against infection caused by intracellular pathogens such as viruses and certain bacteria to function as a Th1 type cytokine [1-3]. In addition, IFN-γ also regulates cell growth affecting differentiation, survival, and apoptosis in a wide range of cell types [4]. These actions of IFN-γ are shown to be mediated by a large number of IFN-γ-induced specific gene products which include interferon regulatory factors [5,6], antiviral factors [7-9], chemokines [10,11], cytokine receptors [12], signaling molecules [13,14], and apoptosis-regulatory factors [15,16]. As a part of our ongoing investigation of the mechanisms involved in regulation of the Th1 and Th2 immune response, we screened for novel target genes whose expressions are differentially regulated by Th1 and Th2 cytokines by performing differential display-polymerase chain reaction (DD-PCR) analysis with human peripheral blood mononuclear cells (PBMCs). From such analysis human thioredoxin (Trx-1) was identified as a novel target specifically induced by IFN-γ.

Mammalian thioredoxins are a family of proteins that contain a conserved -Trp-Cys-Gly-Pro-Cys-Lys- catalytic site. When combined with glutathione, thioredoxins constitute a major group of redox proteins responsible for the regulation of intracellular redox status [17,18]. During the redox regulation, thioredoxin undergoes reversible oxidation/reduction of the two cysteine groups. The dithiol(-SH) form of thioredoxin reduces oxidized protein substrates that contain a disulfide group, and the oxidized form then cycles back in an NADPH-dependent process that is mediated by thioredoxin reductase, another protein that contains a thiol group [19,20]. Thioredoxin is released from the cell in a redox-sensitive manner, and the serum thioredoxin level is considered to be an indicator of oxidative stress, especially in cases of liver diseases [21,22].

It was initially reported that human thioredoxin stimulated the growth of transformed T and B cell lines [23,24]. Since then, it has been suggested that thioredoxin has both apoptotic and survival functions in diverse cell systems [25]. Recently, studies evaluating the anti-apoptotic effect of thioredoxin have indicated that thioredoxin, through its redox-control functions, affects cell growth and survival by perturbation of specific apoptosis signaling molecules, such as apoptosis-stimulating kinase-1 [26,27]. In addition, it has been reported that truncated thioredoxin (Trx80) stimulates monocytes/macrophages to induce IL-12, implying that it is involved in immune-inflammatory reactions that direct Th1 immunity and IFN-γ production [28].

In light of these findings which suggest that thioredoxin functions in the regulation of immune cell growth and possibly in Th1 immune response, it was interesting for us to identify thioredoxin as a novel target induced by IFN-γ in cells of immunological origin. Therefore, we examined the mechanism by which IFN-γ induces and regulates thioredoxin gene expression. In addition, we evaluated the role that thioredoxin plays in immune cell survival and cytokine production upon oxidative stress. The results of this study shed light on the coordinated immune defense function of IFN-γ and thioredoxin during diverse stress responses to infection and apoptotic stimuli.

## Results

### 1. Identification of thioredoxin as a novel target induced by IFN-γ

During DD-PCR screening for novel factors involved in the modulation of Th1 and Th2 immune response, we identified a number of target genes that were differentially regulated by Th1 and Th2 cytokines [29,30]. In particular, by screening mRNAs isolated from human PBMCs stimulated with IL-4 and/or IFN-γ by DD-PCR, Clone A1 was first noted as a product selectively induced upon stimulation by IL-4 and IFN-γ, but not by IL-4 alone, indicating that it is an IFN-γ-induced factor. By using Clone A1 as a probe on a Northern blot analysis of human PBMCs, a specific induction by IFN-γ of a single mRNA species with 550 bases was detected (see Additional file 1). Subsequent cloning and sequence analysis revealed that Clone A1 contained part of the 3' coding and the 3'UTR sequence of human thioredoxin as in MGC:5174 (Genebank accession # BC003377). Subsequently, screening of Jurkat T cell cDNA expression libraries using the Clone A1 probe and the RT-PCR cloning produced a clone of full-length thioredoxin cDNA that was comprised of 530 bases coding 105 amino acids, including the conserved 31Trp-Cys-Gly-Pro-Cys35 motif (see Additional file 1) which was identical to the previously reported sequence of human thioredoxin [31].

### 2. Regulation of thioredoxin gene expression by IFN-γ in immune cells

**Figure 1:**
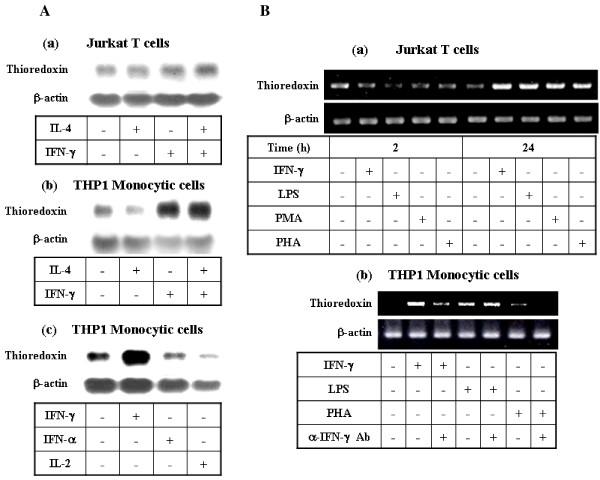
**Regulation of thioredoxin gene expression in immune cell lines**. **A. Regulation of thioredoxin gene expression by cytokines in immune cell lines**. Human lymphocytic T cell line, Jurkat (**Panel a**) and the promonocytic cell line, THP1 (**Panels b and c**), were maintained in complete RPMI media. Cells (5 × 10^6^) were then treated with various cytokines (10 ng/ml IFN-γ, 10 ng/ml IL-4, 10000 u/ml IFN-α, and 10 ng/ml IL-2) as indicated for 24 h. The total RNA was then isolated and analyzed by Northern blot using a full-length cDNA probe of human thioredoxin. The membranes were stripped and reprobed for β-actin as an internal control. **B. Effect of IFN-γ and mitogens on thioredoxin gene expression. Panel a**: Jurkat T cells (2 × 10^6^) were treated with IFN-γ (10 ng/ml), LPS (1 μg/ml), PMA (10 ng/ml) or PHA (2.5 μg/ml) in serum-free media for the indicated durations, after which RNAs were isolated and analyzed by RT-PCR using primers specific for thioredoxin. **Panel b**: THP1 monocytic cells (2 × 10^6^) were treated with IFN-γ (10 ng/ml), LPS (1 μg/ml), or PHA (2.5 μg/ml) in the presence or absence of anti-IFN-γ Ab (10 μg/ml). The cells were then cultured for 24 h, after which the total RNA was isolated and analyzed by RT-PCR using primers specific for thioredoxin and IFN-γ.

Using a full-length cDNA probe for thioredoxin, the induction and regulation of human thioredoxin gene expression by IFN-γ was examined in various immune cells. Northern blot analysis revealed that IFN-γ significantly up-regulated the thioredoxin mRNA levels in THP1 monocytic cell line and Jurkat T cell line, although the up-regulation was less significant in Jurkat cells. Conversely, IL-4, a prototypic Th2 cytokine, did not have an inducing effect on the thioredoxin mRNA levels in these cells (Fig 1A-a, b). RT-PCR analysis also revealed a similar regulation pattern of thioredoxin gene expression in IFN-γ- or IL-4-treated THP1 and Jurkat cells. (see Additional file 2). The thioredoxin-inducing effect of IFN-γ was also seen in Ramos B cells and human PBMCs [32]. Taken together, these data indicate that the induction of thioredoxin gene expression by IFN-γ is common to various types of immune cells.

Prior studies evaluating human thioredoxin mRNA expression and regulation in immuno-competent cells have shown that, although the constitutive level of thioredoxin expression is relatively low in primary lymphocytes and monocytes, its level is significantly up-regulated in these cells upon activation by mitogens such as PMA, PHA, and LPS [31,33]. In fact, we observed the induction of thioredoxin by PMA, PHA, and LPS in Jurkat T cells and THP1 cells in this study (Fig 1B-a, b). However, the mechanisms by which thioredoxin induction occurs in response to these mitogens appear to differ, as indicated by the differential effects of anti-IFN-γ antibodies on thioredoxin gene expression induced by LPS and PHA. Specifically, treatment of cells with anti-IFN-γ suppressed the thioredoxin-inducing effect of PHA, suggesting that PHA-induced thioredoxin expression is mediated by the action of IFN-γ (Fig 1B-b).

While IFN-γ induced prominent up-regulation of thioredoxin in monocytic cells, neither IL-2 nor IFN-α had a significant enhancing effect on thioredoxin expression in these cells (Fig 1A-c). The up-regulation of thioredoxin by IFN-γ observed in monocytic cells may have an important functional implication, since mononuclear phagocytes are the primary target of IFN-γ during the immune and inflammatory responses that involve redox-regulatory mechanisms during the respiratory burst [34]. Therefore, we evaluated the mechanism by which thioredoxin induction by IFN-γ occurred in monocytic cells using the THP1 cell line.

### 3. Induction and regulation mechanism of thioredoxin expression by IFN-γ

**Figure 2:**
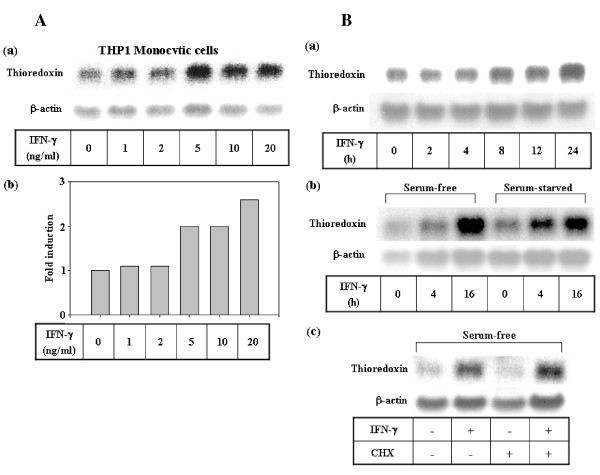
**Up-regulation of thioredoxin gene expression by IFN-γ in monocytic cells**. **A. Dose-dependent induction of thioredoxin mRNA by IFN-γ**. THP1 monocytic cells (5 × 10^6^) were treated with varying doses of IFN-γ in complete media for 24 h, after which the RNA was isolated and the thioredoxin gene expression was analyzed by Northern blot analysis as described in Fig 1. **Panel a**: Northern blot autoradiogram. **Panel b**: Densitometric analysis of the data in panel a. The values represent the fold induction of thioredoxin mRNA levels, which were calculated after normalization against β-actin by taking the untreated control value as 1. **B. Kinetics of thioredoxin gene induction by IFN-γ and the effect of cycloheximide. Panel a**: THP1 cells were treated with IFN-γ (10 ng/ml) in complete media for the indicated time periods, after which the RNA was isolated and analyzed for thioredoxin gene expression by Northern blot analysis as described in Fig 1. **Panel b**: THP1 cells that had been maintained in complete media were washed thoroughly and then treated with IFN-γ (10 ng/ml) under serum-free conditions ("serum-free") or serum starved conditions for 48 h prior to IFN-γ treatment ("serum-starved"). The cells were then harvested after the indicated time periods, after which their thioredoxin mRNA levels were determined by Northern blot analysis. **Panel c**: THP1 cells were treated with IFN-γ under serum-free conditions as described for panel b, with or without pretreatment with cycloheximide (30 μg/ml) for 3 h. The cells were then cultured for an additional 4 h following IFN-γ treatment, after which the thioredoxin mRNA levels were determined by Northern blot.

**Figure 3:**
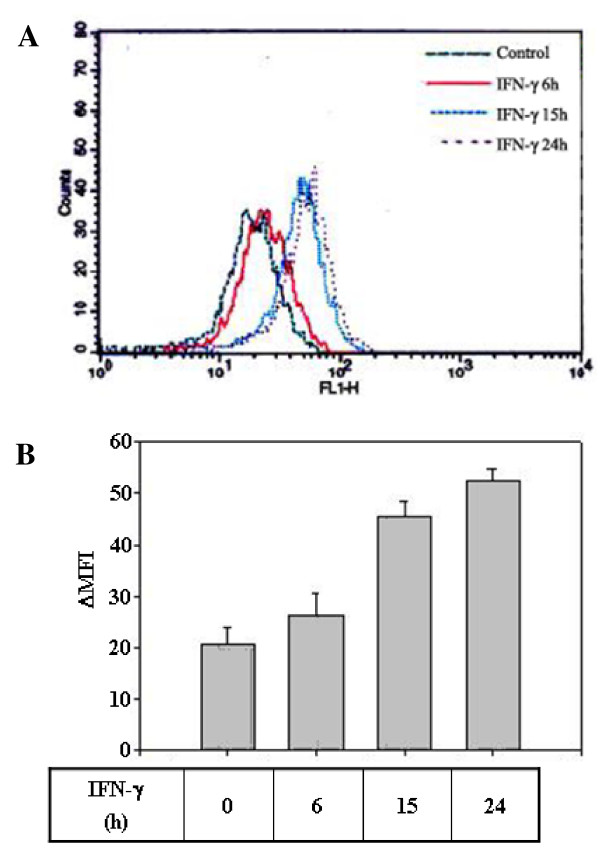
**Up-regulation of the thioredoxin protein levels by IFN-γ**. THP1 cells (5 × 10^5^) were treated with media alone or with IFN-γ under serum-free conditions for the indicated time periods. The cells were then treated with brefeldin A (10 μg/ml) for 4 h prior to being harvested, after which they were permeabilized and stained with mouse monoclonal anti-thioredoxin antibodies and mouse IgG-FITC as described in the text. FACS analysis was performed to determine the intracellular protein levels based on their MFI values. **Panel A**: A representative FACS histogram. **Panel B**: ΔMFI values for thioredoxin levels. Each value shown represents the mean of three independent determinations.

To confirm the specificity of thioredoxin gene regulation by IFN-γ, we examined the dose-dependency and kinetics of thioredoxin mRNA induction by IFN-γ. Treatment of THP1 cells with increasing doses of IFN-γ from 1 to 20 ng/ml resulted in up to a 2.7 fold increase in thioredoxin mRNA levels in a dose-dependent manner (Fig 2A). Furthermore, kinetic analysis of the thioredoxin mRNA induction revealed that, although the mRNA level gradually increases with time in response to IFN-γ treatment both in the presence and absence of serum, a more prominent induction was noted under serum-starved conditions. The thioredoxin induction occurred within 4 h and significantly increased by 16 h post IFN-γ treatment under serum-free conditions (Fig 2B, panels a and b). Moreover, this up-regulation of thioredoxin mRNA levels by IFN-γ was not inhibited by the translational inhibitor, cycloheximide, which suggests that the IFN-γ-mediated induction of thioredoxin is a primary response which does not require on-going protein synthesis (Fig 2B, panel c). The induction of thioredoxin by IFN-γ was also observed at the protein level which kinetically followed the induction of thioredoxin mRNA production. This increase in protein level began within 6 h, and then gradually accumulated for 24 h, ultimately increasing by the same magnitude (2.7 fold) as the mRNA (Fig 3). Taken together, these data indicate that IFN-γ up-regulates the thioredoxin protein levels in monocytic cells through the induction of gene expression.

**Figure 4:**
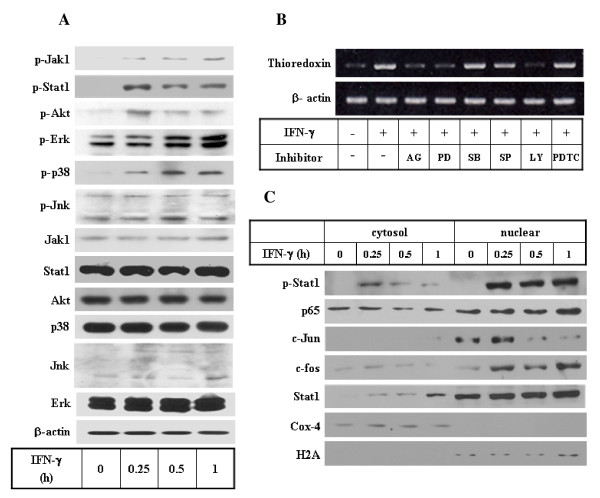
**Analysis of signaling pathways activated by IFN-γ and the effects of signaling inhibitors on thioredoxin gene expression**. **A. Analysis of Jak, Akt, and MAPK pathways during IFN-γ signal transduction in THP1 cells**. THP1 cells (1 × 10^6^) were treated with media alone or IFN-γ under serum-free conditions for the indicated times. The total cell lysates were then prepared and analyzed by Western blot to determine the activation status of Jak1/Stat1, Akt, and MAPKs as described in the text. **B. Effects of signaling inhibitors on IFN-γ-induced thioredoxin gene expression**. THP1 cells (1 × 10^6^) were pretreated with LY294002 (20 μM), PD98059 (50 μM), SP600125 (10 μM), AG490 (10 μM), PDTC (100 μM) or SB203580 (20 μM) for 1 h and then cultured for an additional 24 h in the presence or absence of IFN-γ under serum-free conditions. The total RNA was isolated and the thioredoxin mRNA levels were then analyzed by RT-PCR. **C. Analysis of activation and nuclear translocation of Stat1, NF-κB, and AP-1 induced by IFN-γ in THP1 cells**. Cells (5 × 10^6^) were treated with media alone or IFN-γ (10 ng/ml) under serum-free conditions for the indicated times. The cells were then harvested and used to prepare cytoplasmic or nuclear extracts. The extracts were then subjected to Western blot to determine the activation status of Stat1, c-Jun, c-fos or NF-κB, using antibodies to anti-phosphotyrosine Stat1, anti-Stat1, c-Jun, c-fos and NF-κB (p65). As internal controls, Cox-4 and H2A were used for cytosolic and nuclear marker proteins, respectively.

Because the up-regulation of thioredoxin gene expression appeared to occur as a direct response to IFN-γ without involving on-going protein synthesis in IFN-γ-treated cells, we evaluated the initial signaling pathways involved in the IFN-γ-mediated induction of thioredoxin gene expression. The analysis of the signal pathways induced by IFN-γ in monocytic cells revealed that, in addition to the well-known Jak1/Stat1 pathways [35], IFN-γ generates strong activating signals for Akt, Erk and p38 MAPK within 15 to 60 min (Fig 4A). Treatment with inhibitors of Jak tyrosine kinases (AG490), PI3K/Akt (LY294002) and Erk (PD98059) suppressed the IFN-γ-induced effect on thioredoxin mRNA levels, but treatment with inhibitors of p38 MAPK (SB203580) and Jnk (SP600125) did not (Fig 4B). Additionally, use of a broad-spectrum tyrosine kinase inhibitor, tyrophostin, produced a similar inhibitory effect (data not shown). These results suggest that the early activation of Jak/Stat1, PI3K/Akt and Erk, but not p38 or Jnk, is involved in the IFN-γ-mediated induction of thioredoxin gene expression. Next, we examined the downstream effectors of these kinases which might act as transcriptional activators for thioredoxin gene expression. In addition to Stat1 which acts as a key transcription factor during IFN-γ signaling, AP-1(c-fos/c-jun) and NF-κB were chosen for analysis because these factors were implicated in the regulation of gene expression involved in redox response. The nuclear translocation profile indicated that there was a strong activation of Stat1 and c-jun/c-fos within 15 min and a weak activation of p65/NF-κB within 30 to 60 min upon IFN-γ treatment (Fig. 4C). The activation of Stat1 and AP-1 may occur through Jak1- and Erk/Akt-dependent phosphorylation, respectively. Despite a weak activation of p65/NF-κB by IFN-γ in these cells, its involvement in thioredoxin gene expression is unlikely, since PDTC (an NF-κB inhibitor) had no effects on the IFN-γ-induced thioredoxin mRNA levels. Hence, the data suggest that Stat1 and AP-1, but not NF-κB may play a role in IFN-γ signaling pathways which lead to thioredoxin gene expression.

### 4. Role of thioredoxin in the oxidant- or irradiation-induced stress response to regulate immune cell survival from apoptosis mediated byROS

**Figure 5:**
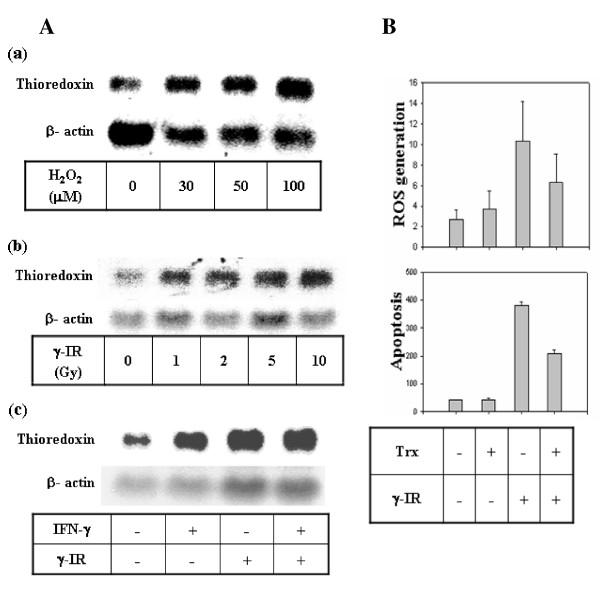
**Role of thioredoxin in the oxidant- or gamma irradiation-induced stress response and apoptosis**. **A. Induction of thioredoxin gene expression by oxidants and gamma irradiation**. THP1 monocytic cells were treated with media alone or with varying doses of hydrogen peroxide for 24 h (**Panel a**). Cells were exposed to varying doses of gamma irradiation (γ-IR) and further cultured for 16 h (**Panel b**). Cells were treated with media alone, IFN-γ (10 ng/ml), γ-IR (5 Gy), and IFN-γ plus γ-IR for 16 h (**Panel c**). Total RNA was prepared and analyzed by Northern blot to determine the thioredoxin mRNA levels. **B. Protective function of thioredoxin against gamma irradiation-induced apoptosis**. U937 monocytic cells were either mock-transfected with empty vector (-) or transfected with thioredoxin expression construct (+) and then cultured for 40 h. The cells were then exposed to 50 Gy of γ-IR, after which they were analyzed for ROS generation at 30 min (upper box) and apoptosis at 16 h (lower box) as described in the text.

One of the most recognized functions of thioredoxin is its ability to increase cellular reductive power and reduce proteins that have been damaged by oxidative stress or various ROS-inducing stimuli [36]. Therefore, we first evaluated the effect of ROS-inducing stimuli on thioredoxin gene expression and compared these effects with those of IFN-γ. As indicated in previous studies [36], hydrogen peroxide or gamma irradiation at sub-lethal doses induced a noticeable up-regulation of thioredoxin gene expression within 24 h (Fig 5A-a, b). In addition, the magnitude of thioredoxin induction that occurred in response to both IFN-γ and irradiation treatment was comparable (2.0~2.5 fold); however, no additive or synergistic effects were observed upon combined treatment. These findings suggest that IFN-γ and gamma irradiation induce thioredoxin through a common or converging pathway (Fig 5A-c).

To examine the role that thioredoxin plays in immune cell survival during apoptotic stress, we analyzed the effect of thioredoxin expression on the apoptosis induced by oxidative or radiation stress. We found that monocytic cells generated ROS and showed an apoptotic response upon exposure to 50 Gy of γ-irradiation, and that transfection of thioredoxin substantially suppressed the ROS levels and partially protected the cells from the irradiation-induced apoptosis (Fig 5B). Similarly, apoptosis induced by hydrogen peroxide was substantially suppressed upon forced expression of thioredoxin in target cells (data not shown). The results are in agreement with the study conducted by Ferret et al. [37], which demonstrated that transfected thioredoxin protected THP1 monocytic cells from NO-mediated cellular injury. Together these data indicate that human thioredoxin plays a role in immune cell survival upon exposure of cells to ROS-inducing stimuli, such as hydrogen peroxide or gamma irradiation.

### 5. Regulation of cytokine balance by thioredoxin through the induction of IFN-γ during oxidative stress

**Figure 6:**
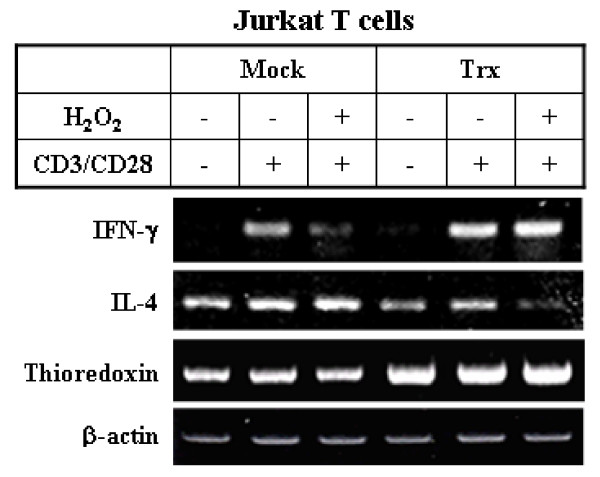
**The effects of oxidants and thioredoxin on cytokine production in Jurkat T cells: Thioredoxin up-regulates IFN-γ production and counteracts Th2 cytokine bias caused by oxidants**. Jurkat T cells were either mock-transfected with empty vector (Mock) or transfected with thioredoxin expression construct (Trx) and then incubated for 24 h. The cells were then pretreated with hydrogen peroxide (400 μM) for 1 h, after which they were incubated with media alone or with human anti-CD3 (1 μg/ml) plus anti-CD28 (1 μg/ml) to stimulate cytokine production for 4 h. RT-PCR was then performed to determine the mRNA levels of IL-4, IFN-γ, and thioredoxin.

To assess the immunological functions of IFN-γ-induced thioredoxin during oxidative stress, we examined the effect of thioredoxin on T cell cytokine balance. It has been reported that exogenously added Trx80 stimulates IL-12 production and enhances CD14 expression on monocytes in PBMC cultures [28]. In addition, it has been suggested that the intracellular thiol redox status of macrophages plays a role in the regulation of cytokine balance towards Th1 in thioredoxin-transgenic mice during aging [38]. Conversely, several studies have shown that oxidative stress and ROS signaling tend to increase Th2 cytokine production and induce a Th2-biased cytokine profile [39,40]. Therefore, we evaluated the effect of thioredoxin to determine if it regulates the Th1/Th2 cytokine balance during oxidative stress. It was observed that stimulation with anti-CD3 plus anti-CD28 effectively induced both IL-4 and IFN-γ expression in cytokine-producing Jurkat T cells and that hydrogen peroxide treatment selectively suppressed the IFN-γ gene expression induced by anti-CD3 plus anti-CD28, resulting in the cytokine balance leaning towards Th2 (Fig 6, lane 2 vs. lane 3). Noticeably, in Jurkat T cells that were transfected with thioredoxin, the IFN-γ mRNA levels were upregulated, whereas the IL-4 mRNA levels were down-regulated (Fig 6, lane 3 vs. lane 6). These results demonstrate that the oxidant-induced Th2-biased cytokine response is counteracted by thioredoxin, which suggests that thioredoxin plays a role in Th1 immunity through the induction of IFN-γ.

**Figure 7:**
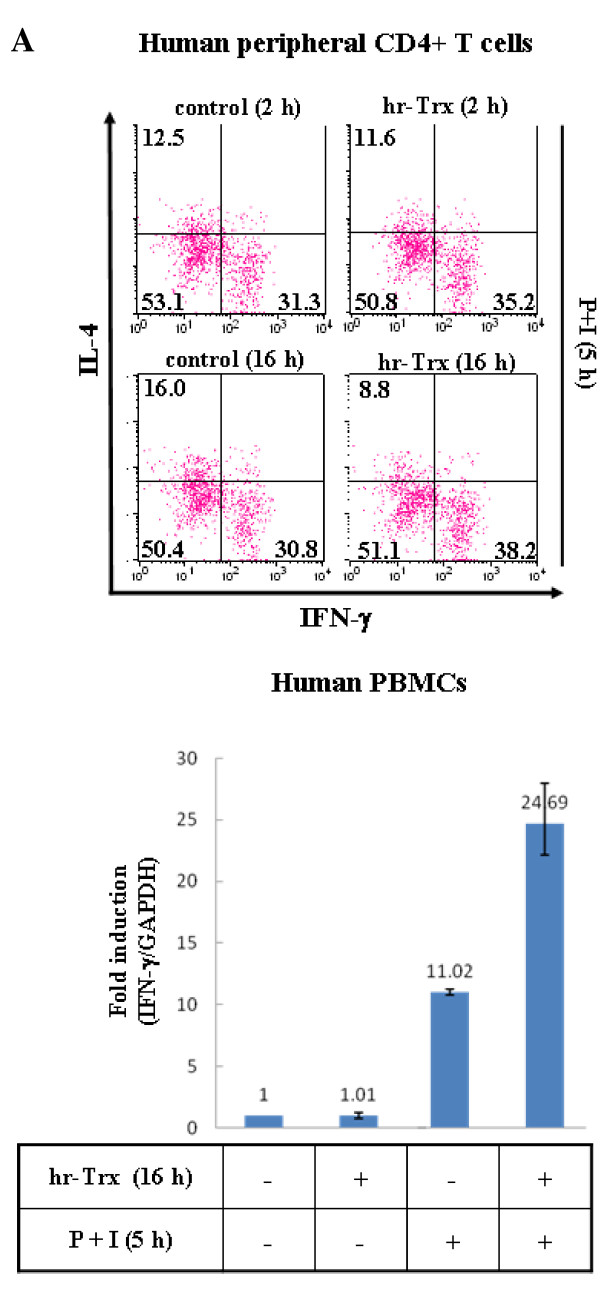
**Effects of recombinant thioredoxin (hr-Trx) on cytokine production in primary immune cells**. **A. Thioredoxin modulates IFN-γ- vs. IL-4-producing CD4+ T cell populations**. Human peripheral lymphocytes (1 × 10^6^) were treated with media alone or with human recombinant thioredoxin (hr-Trx) for 2 or 16 h. The cells were then stimulated with PMA (30 ng/ml) + ionomycin (1 μg/ml) in the presence of brefeldin A (10 μg/ml) for 5 h, after which they were permeabilized and stained with FITC-conjugated anti-human IFN-γ antibodies and PE-conjugated anti-human IL-4 antibodies. These cells were then restained with PerCP-conjugated anti-human CD4 antibodies to enable separation of the Th cells. FACS analysis was performed to determine the intracellular cytokine levels. **B. Thioredoxin increases the IFN-γ mRNA levels in human PBMCs**. Human PBMCs (1 × 10^6^) were treated with media alone or with hr-Trx for 16 h. The cells were then stimulated with PMA (20 ng/ml) + ionomycin (0.5 μg/ml) for 5 h. The total RNA was then isolated and analyzed by real time RT-PCR using primers specific for IFN-γ. IFN-γ mRNA expression levels were normalized with the internal control, GAPDH.

Next, in order to investigate the role of thioredoxin on cytokine production in primary immune cells, we treated human PBMCs with recombinant human thioredoxin (hrTrx) and then analyzed the cytokine-producing cell populations for IFN-γ and IL-4 using intracellular cytokine staining (Fig 7A). Upon hrTrx treatment, mitogen-induced IFN-γ-producing cells were increased (30.8 vs. 38.2%), while IL-4-producing cells were decreased (16.0 vs. 8.8%), which resulted in a clear shift towards Thl. Furthermore, analysis by quantitative RT-PCR revealed that, in accordance with the increase in IFN-γ-producing cells, hrTrx treatment significantly up-regulated the mitogen-induced IFN-γ mRNA levels by 2-fold (Fig 7B).

## Discussion

As a part of our on-going investigation on the regulation mechanism involved in Th1 and Th2 immune response, we conducted DD-PCR analysis of human PBMCs to screen for novel target genes differentially regulated by Th1 and Th2 cytokines. The results of our screening revealed that human thioredoxin was selectively induced by IFN-γ, but not by IL-4, in various immune cells. Human thioredoxin was originally identified as a soluble factor produced by HTLV- or EBV-transformed lymphoblastoid cell lines which stimulated the growth of these cell lines [24]. While the constitutive levels of thioredoxin were diverse depending on the cell types [31,33], relatively low thioredoxin mRNA expression levels in immune cells were generally enhanced upon mitogen or oxidant treatment. We have also noted that various mitogens were capable of thioredoxin induction in T and monocytic cells within 24 h. More importantly, we have demonstrated that thioredoxin is up-regulated up to 3 fold by a prototypic Th1 cytokine IFN-γ in various immune cell types including PBMCs and B cells [32] as well as T and monocytic cells (Fig 1). In monocytic cells, the thioredoxin-inducing effect is specific to IFN-γ, while other cytokines such as IL-2 and IFN-α, had no effect on thioredoxin expression (Fig 1A and 1B). The prior incubation of cells with neutralizing antibodies to human IFN-γ suppressed the increase in thioredoxin mRNA levels that was induced by IFN-γ and PHA, but not the increase caused by LPS, suggesting that PHA-induced thioredoxin expression is mediated by IFN-γ (Fig 1B). The up-regulation of thioredoxin gene expression in monocytic cells was more prominent under serum-free conditions than serum-rich conditions (Fig 2B), which suggests that growth factors contained in the serum may affect the regulation of thioredoxin levels.

To elucidate the regulation mechanism by which the thioredoxin gene is induced by IFN-γ, we conducted a kinetic analysis and examined the signaling pathways involved in regulation of gene using a monocytic cell line. The increase in mRNA levels induced by IFN-γ was kinetically followed by increased production of thioredoxin protein, and the same magnitude of induction was obtained at the mRNA and protein levels. These findings indicate that IFN-γ-induced thioredoxin gene expression leads to increased protein production. The up-regulation of thioredoxin mRNA by IFN-γ was not affected by cycloheximide, a translational inhibitor. This observation suggests that the induction of thioredoxin is a primary response to IFN-γ, which does not require on-going protein synthesis or other IFN-γ-induced protein products. Therefore, the early IFN-γ signaling pathways would directly influence the activation of transcription factors responsible for the thioredoxin gene expression. The subsequent analysis of the IFN-γ-activated signaling transduction pathways and the subsequent experiments evaluating the effects of specific signaling inhibitors showed that the IFN-γ-induced up-regulation of the thioredoxin gene expression involves Jak, PI3k/Akt and Erk-dependent pathways, which lead to the activation of specific transcription factors.

Although several transcription factors have been implicated in the regulation of thioredoxin gene expression in response to oxidative stress, including Ap-1/Ref, NF-κB, and ATFII, relatively little is known about the mechanism by which these transcription factors are activated, their binding to responsive elements, or the eventual transcriptional activation of the thioredoxin gene [41,42]. However, a close examination of the human thioredoxin gene promoter sequence revealed the presence of putative binding sites for the Stat proteins (GAS). This observation together with the data that early activation of Jak1/Stat1 during IFN-γ signaling and the effect of Jak inhibitors on thioredoxin gene expression, strongly suggest that tyrosine-phophosphorylated Stat1 plays a direct role in the stimulation of IFN-γ-induced thioredoxin gene expression (Fig 4-A, B, and 4C). In addition, IFN-γ induced early nuclear translocation of c-jun and c-fos, and a weak delayed activation of NF-κB (Fig 4C). The activation of AP-1 and NF-κB by Erk and PI3K/Akt-dependent phosphorylation have been suggested [43,44], which correlates with the result in Fig 4-B. However, the role of these transcription factors in IFN-γ-activated thioredoxin gene expression needs to be investigated in more detail in future studies.

One of the factors recently found to play a key role in antioxidant response is nuclear factor-erythroid 2-related factor (Nrf). Nrf binds to the antioxidant response element (ARE) present in the promoter of detoxifying or anti-oxidant enzymes such as heme oxygenase-1, glutathione-S-transferase, thioredoxin reductase, and thioredoxin [45]. Because the Nrf binding site located on the thioredoxin promoter overlaps the AP-1 site [46], it is possible that the AP-1 activating signal of IFN-γ replaces the transcriptional activation of the thioredoxin gene via the Nrf/ARE element. However, few studies evaluating the regulation mechanism of thioredoxin gene expression by cytokines or growth factors have been conducted to date. Therefore, detailed studies evaluating the IFN-γ signaling pathways that lead to transcriptional activation of the thioredoxin gene should be performed to provide information regarding the mechanism by which transcriptional regulation of thioredoxin expression occurs.

The induction of thioredoxin expression by IFN-γ noted in various immune cells in our study may have an important functional implication. We found that sub-lethal doses of hydrogen peroxide or gamma irradiation induced thioredoxin, but that this induction was delayed (Fig 5). This finding is in agreement with the well-recognized roles that thioredoxin plays in defense against oxidants and radiation stress generating ROS. However, the administration of lethally high doses of these agents often leads to the early suppression of thioredoxin levels and subsequent cell death (data not shown). In our study, IFN-γ-induced thioredoxin gene expression occurred earlier (4 h; Fig 2) than oxidative- or radiation stress-mediated induction of thioredoxin (16–24 h), and co-treatment with IFN-γ and irradiation stress did not exert a synergistic effect on the induction of thioredoxin (Fig 5A). Taken together, these observations suggest that gamma irradiation and IFN-γ induce thioredoxin expression through a common pathway, and that IFN-γ acts as a mediator of irradiation-induced thioredoxin up-regulation.

Thioredoxin has long been recognized for its ability to protect cells from apoptotic stress. For example, in a murine lymphoid cell line WEHI7.2, the transfection and over-expression of human thioredoxin was found to suppress the apoptosis induced by glucocorticoid, etoposide, and peroxide, in a manner similar to Bcl2 over-expression [47]. However, the mechanism by which thioredoxin exerts its anti-apoptotic effect against different stress responses is not clearly known, while it appears to vary depending on the nature of the stimuli and the types of cells involved [48,49]. However, results of our study indicates that thioredoxin over-expression suppresses the irradiation- or oxidant-induced apoptosis in immune cell lines, and that such anti-apoptotic function of thioredoxin is associated with the ROS-scavenging effect observed in thioredoxin-transfected cells.

The redox-regulation of mononuclear phagocytes plays an important physiological role in the defense against microbial infection and inflammation, as well as in the proper cellular response to a wide variety of stress conditions evoked by hypoxia/oxidants, nutrient starvation, DNA damaging agents, and ionizing radiation [50]. It is now apparent that thioredoxin is a key defense molecule involved in the regulation of homeostasis in cells subjected to diverse stresses [19,50]. On the other hand, the functions of IFN-γ as an immuno-regulatory cytokine include macrophage activation for effective antigen processing and presentation upon pathogen uptake via up-regulation of MHC molecules [51], activation of lysosomal enzymes [52], and regulation of ROS-generating enzymes such as NADPH oxidases [53,54], myeloperoxidases [55] and superoxide dismutases [56]. Recently, an IFN-γ-inducible thiol-containing lysosomal enzyme, GILT, which plays a role in the control of oxido-reduction reactions in the lysosome during antigen processing, was identified [57,58]. These findings indicate that there is a casual mechanistic link between redox regulation of the thioredoxin system and IFN-γ-mediated macrophage activation, which functions as an important defense mechanism that maintains immune homeostasis upon infection and inflammation.

It has been reported that mammalian thioredoxins are produced in several forms different in size and structure. In addition to the classical cytosolic Trx-1 [31] and mitochondrial Trx-2 [59], a minor cytosolic TRP14 [60] and Trx80 [61] have been identified. The 12 kD Trx-1 and C-terminal truncated 10 kD Trx80 are known to be secreted from the cell and believed to act as a co-cytokine to regulate immune cell functions [61,62]. Denaturing SDS-PAGE analysis of IFN-γ-treated THP-1 cells revealed that the IFN-γ-induced thioredoxin identified in the present study has a deduced molecular weight of 12 kD (Fig S1) and an apparent molecular weight of 14 kD (data not shown), which indicate that it is the cytosolic full-length Trx-1 [31]. The reported cytokine-like activities of both full-length and truncated thioredoxin include stimulation of immune cell proliferation, macrophage differentiation and chemotaxis, and production of inflammatory cytokines such as IL-I, IL-6, IL-8, and TNF [63-65].

Of particular interest is the finding that Trx80, when co-treated with IL-2, promoted the production of IL-12 and IFN-γ by human PBMCs [28], which suggests that thioredoxin can act as a potential inducer of Th1 immunity and that there is a functional link between thioredoxin and IFN-γ. In line with this, our data demonstrate that thioredoxin over-expression induces T cells to counteract the oxidant-induced Th2 bias and reverse cytokine balance by up-regulating IFN-γ gene expression (Fig 6). Furthermore, a Th1-inducing effect of thioredoxin was observed in human primary CD4+ T cells, as shown by changes between the IL-4 and IFN-γ-producing populations in response to treatment with hrTrx (Fig 7A). Specific induction of IFN-γ mRNA in PBMCs treated with hrTrx further supported the up-regulation of Th1 response by thioredoxin (Fig 7B). The induction of thioredoxin by IFN-γ and the restoration of IFN-γ levels by thioredoxin during oxidative stress in immune cells found in this study, suggest that thioredoxin and IFN-γ may have evolved to serve complementary functions through positive feedback mechanisms during immune inflammatory conditions.

## Conclusion

We have demonstrated that human thioredoxin is a novel target gene induced by IFN-γ. In addition, we showed that thioredoxin may play a role in the protection of immune cells from apoptosis and in the counter-regulation of cytokine production evoked by ROS-generating stimuli. Therefore, thioredoxin induced by IFN-γ upon infection, stress, or immune triggers for Th1 immune response appears to act as a protective factor that maintains immune cell homeostasis through mechanisms involving ROS-scavenging, anti-apoptotic and cytokine-regulating functions.

## Methods

### Cells and cell culture

PBMCs were isolated from the freshly-drawn blood of healthy donors using Ficoll-Hypaque density gradient centrifugation. Flow cytometry revealed that the mononuclear cell preparation typically contained approximately 70% T cells, 15~20% B cells, and 10~15% monocytes. The human monocytic cell lines, THP1 and U937, and the T lymphocytic leukemic cell line, Jurkat, were obtained from ATCC and maintained in complete RPMI media containing 10 mM Hepes (pH 7.5), 50 μM β-mercaptoethanol, and 10% FBS (Life Technologies Inc., Grand Island, NY USA). The cells were then treated with recombinant human IFN-γ, IL-4, IL-2 (R & D systems, Minneapolis, MN, USA) or IFN-α (Roche, Nutley, NJ USA) in the presence or absence of serum, after which the cells were cultured for various lengths of time at 37°C under 5% CO_2_.

### DD-PCR screening and cloning

PBMCs were cultured in the presence of 10 ng/ml of IFN-γ and/or IL-4, after which the total RNA was isolated using 4 M GITC and 5 M CsCl and ultracentrifugation. DD-PCR assays were performed using a DD-PCR kit (RNAimage, GenHunter, Nashville, USA) according to the manufacturer's instructions, and the amplified products were displayed on a 6% polyacrylamide gel. Next, PCR product bands that were selected based on their differential expression were purified from the gel, reamplified, and ligated into a pUC18/*Sma*I vector at 16°C for 48 h. E. *Coli *was then transformed with the ligated vectors, after which clones that contained the insert were selected. The plasmids were then purified and sequenced using a previously described method [29].

### Northern blot and RT-PCR analysis of thioredoxin

Cells were treated with various cytokines for the indicated times, after which the total RNA was isolated. Next, the RNA was separated on a 1% agarose gel, transferred to nylon membranes, and then hybridized with a radio-labeled probe of a full-length human thioredoxin cDNA. Densitometric analysis of the blots was then performed using ImageQuant (Molecular Dynamics, Sunnyvale, CA, USA). RT-PCR analysis of thioredoxin was performed using standard protocols. Briefly, RNAs (1~2 μg) were reverse-transcribed and then subjected to 30 cycles of PCR amplification using a thermal cycler (GeneAmp PCR System 2400, Applied Biosystems, USA) and the following thioredoxin specific primers: 5' primer, CTTTGGATCCATTTCCATC; 3' primer, GCATTAATGTTTTATTGTCACG., as described [32].

### Intracellular staining of thioredoxin

Cells were cultured in serum-free medium and then treated with IFN-γ for various time periods. To measure the intracellular levels of thioredoxin, brefeldin A (Sigma, St. Louis, MO, USA) was added to the cells 4 to 6 h prior to harvest. Cells were then permeabilized in PBS buffer containing 0.5% saponin and 1% BSA, after which they were stained with mouse monoclonal anti-human thioredoxin antibodies (American Diagnostics Inc., Stamford, Connecticut, USA) and anti mouse IgG-FITC. Flow cytometric analysis using a FACS Calibur (BD Bioscience, Mountain View, CA, USA) was then conducted to determine the mean fluorescence intensity (MFI) values. The ΔMFI for each sample was then calculated by subtracting the MFI of cells that were stained with anti-thioredoxin antibodies and anti-mouse IgG-FITC from the MFI of cells that were stained with anti-mouse IgG-FITC only. All values represent a mean of three independent determinations.

### Thioredoxin transfection

Cells were plated at a density of 5 × 10^6^/well in 6-well plates. cDNA expression constructs for human thioredoxin, hTRX-WT (kindly provided by Dr. J. Yodoi, Kyoto Univ.) cloned into pcDNA Xpress vector or empty vectors were then introduced into the cells using Superfect reagent (Qiagen, Valencia, USA) according to the manufacturer's instructions. At two days (40 to 48 h) after transfection, the cells were harvested and the thioredoxin expression levels were determined by immunoblotting using monoclonal anti-human thioredoxin antibodies.

### Immunoblotting

Cells were harvested and the total, cytosolic, or nuclear extracts were then prepared. Next, the proteins were fractionated by SDS-PAGE, transferred to PVDF membranes, and then subjected to immunoblot analysis using antibodies to thioredoxin (American Diagnostica Inc.), p65 NF-κB (Upstate Biotechnology, Lake Placid, NY, USA), H2A, Akt, p-Akt, Erk, p-Erk, Jnk, p-Jnk, p38 and p-p38 (Cell Signaling Technologies, Beverly, MA, USA), Jak1, p-Jak1, Stat1, p-Stat1, c-fos and c-jun (Santa Cruz Biotechnology, Santa Cruz, CA, USA), Cox-4 (Invitrogen, Carlsbad, USA) or β-actin using an ECL detection kit (Amersham, Uppsala, Sweden).

### Apoptosis assays

Cells were treated with 10 to 500 μM hydrogen peroxide or irradiated at room temperature with a ^137^Cs γ-source irradiator at a dose rate of 5.66 Gy/min using an IBL 437 type H irradiator (CIS Biointernational, Nice, France) at 5 to 50 Gys as described previously [66]. The cells were then incubated at 37°C, after which apoptosis was determined by cytochrome C release analysis and Annexin V staining as described [32,67].

### ROS measurement

After exposure of the cells to hydrogen peroxide or gamma irradiation for the indicated time periods, cells were collected and stained with 5 μM each of DHE (Calbiochem, San Diego, CA, USA) and H_2_DCF-DA (Sigma) to enable the detection of superoxide anion and hydrogen peroxide, respectively. The fluorescence was then measured by FACSCalibur analysis. The ROS levels are shown as fluorescence intensity, and all values shown represent the mean of three independent determinations.

### Cytokine gene expression analysis

Jurkat T cells were stimulated with anti-CD3 plus anti-CD28 with or without pretreatment with hydrogen peroxide. Following incubation for 4 h, the total RNA was isolated and used for RT-PCR analysis of the cytokine transcripts using primers specific for human IL-4, IFN-γ, and β-actin as described previously [68].

### Intracellular cytokine staining

Human PBMCs were treated with human recombinant thioredoxin (hr-Trx, BD Biosciences) for 2 or 16 h. For intracellular cytokine staining, the cells were stimulated with 30 ng/ml PMA and 1 μg/ml ionomycin for 5 h in the presence of brefeldin A at 37°C. Next, the cells were fixed and permeabilized in Cytofix/Cytoperm buffer, after which they were stained with FITC-conjugated anti-IFN-γ and PE-conjugated anti-IL-4 (BD Biosciences). To separate the CD4+ T cells, these cells were stained with PerCP-conjugated anti-CD4 (BD Biosciences). Flow cytometric analysis was then performed using FACSCalibur to determine the ratio (%) of cytokine-producing cells.

### Real-time RT-PCR

Human PBMCs were treated with hr-Trx for 16 h and then further cultured with 20 ng/ml PMA and 0.5 μg/ml ionomycin for 5 h. Total RNA (1 μg) was then reverse-transcribed, after which real-time PCR amplification with iQ SYBR Green (Bio-Rad) was performed using a Mastercycler realplex thermalcylcer (Eppendorf AG, Hamburg, Germany). The results were normalized against GAPDH by comparing the fold change in the expression of IFN-γ mRNA to the expression of GAPDH using the 2^ΔΔct ^comparative expression method. The following primers were used: for IFN-γ (198 bases), 5'primer, TCCCATGGGTTGTGTGTTTA; 3' primer, AAGCACCAGGCATGAAATCT; for GAPDH (178 bases): 5' primer, GACATCAAGAAGGTG GTGAA; 3' primer, TGTCATACCAGGAAATGAGC.

## Abbreviations

DD-PCR: differential display-polymerase chain reaction; γ-IR: gamma irradiation; Jak: Janus tyrosine kinase; PBMCs: peripheral blood mononuclear cells; ROS: reactive oxygen species; RT-PCR: reverse transcription based polymerase chain reaction; Stats: signal transducers and activators of transcription; UTR: untranslated region.

## Authors' contributions

The authors' contributions to the present study were as follows: S-HK performed the thioredoxin expression analysis, ICC, and RT-PCR to evaluate the cytokine expression. JO analyzed the IFN-γ signaling pathways and transcription factor activation, and conducted the thioredoxin over-expression experiments. J-YC and J-YJ performed experiments in the initial stage of this work, including DD-PCR, cloning and Northern blot analysis of thioredoxin. M-WK conducted the ROS and apoptosis-related work. C-EL designed the research, analyzed the data, and wrote the manuscript.

## Supplementary Material

Additional file 1 **Identification of human thioredoxin as an IFN-γ-induced target gene.Identification of thioredoxin as an IFN-γ-induced target gene**. **Panel a**: After isolation, PBMCs (1 × 10^7^/sample) were treated with media alone or with 10 ng/ml of IFN-γ and/or IL-4 for 24 h. The total RNA was then isolated and processed for DD-PCR analysis with oligo-dT (H-T_11_A) and arbitrary H-AP6(A) primers using a DD-PCR kit as described in the text [29], after which the products were displayed on 6% polyacrylamide gels. Clone A1 was detected as a specific product induced by IFN-γ treatment. **Panel b**: PBMCs (1 × 10^7^/sample) were treated with media alone, IFN-γ, or IL-4 (10 ng/ml each) as indicated. The total RNA was then isolated and analyzed for mRNA that had hybridized with the labeled Clone A1 probe. A single RNA species that was 550 bases in size was detected. **Panel c**: The nucleotide sequence of Clone A1 corresponds to the 3' portion of human thioredoxin cDNA. Upper panel: Complete nucleotide sequence of human thioredoxin. The Clone A1 sequence is indicated by bold characters. The initiation (ATG) and the termination (TAA) codons are underlined. Lower panel: Amino acid sequence of human thioredoxin showing catalytic redox-sensing CGPC residues. Click here for file

Additional file 2 **RT-PCR analysis of thioredoxin gene expression induced by IFN-γ or IL-4 in immune cell lines.** Jurkat T cells (2 × 10^6^) (**Panel a**) or THP1 monocytic cells (5 × 10^6^) (**Panel b**) were treated with media alone, IL-4, or IFN-γ as in Fig 1-A. The total RNA was then isolated and RT-PCR was performed using primers specific for the full-length thioredoxin cDNA. β-actin was used as an internal control. Click here for file
