# Supplementary figures and images for: Identification of human thioredoxin as a novel IFN-gamma-induced factor: Mechanism of induction and its role in cytokine production
Source: BMC Immunol. 2008 Nov 5;9:64. doi: 10.1186/1471-2172-9-64 (PMC2596082; doi:10.1186/1471-2172-9-64)

**
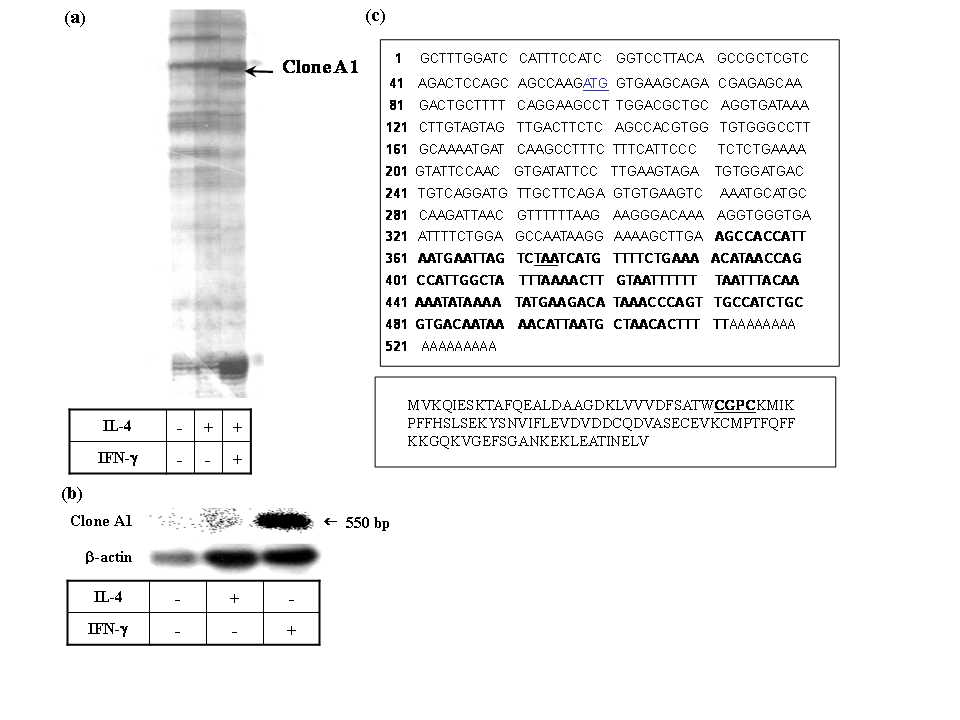
**

Supplement: Additional file 1 — Identification of human thioredoxin as an IFN-γ-induced target gene.Identification of thioredoxin as an IFN-γ-induced target gene. Panel a: After isolation, PBMCs (1 × 10^7^/sample) were treated with media alone or with 10 ng/ml of IFN-γ and/or IL-4 for 24 h. The total RNA was then isolated and processed for DD-PCR analysis with oligo-dT (H-T11A) and arbitrary H-AP6(A) primers using a DD-PCR kit as described in the text [29], after which the products were displayed on 6% polyacrylamide gels. Clone A1 was detected as a specific product induced by IFN-γ treatment. Panel b: PBMCs (1 × 10^7^/sample) were treated with media alone, IFN-γ, or IL-4 (10 ng/ml each) as indicated. The total RNA was then isolated and analyzed for mRNA that had hybridized with the labeled Clone A1 probe. A single RNA species that was 550 bases in size was detected. Panel c: The nucleotide sequence of Clone A1 corresponds to the 3' portion of human thioredoxin cDNA. Upper panel: Complete nucleotide sequence of human thioredoxin. The Clone A1 sequence is indicated by bold characters. The initiation (ATG) and the termination (TAA) codons are underlined. Lower panel: Amino acid sequence of human thioredoxin showing catalytic redox-sensing CGPC residues. [file 1471-2172-9-64-S1.doc]

**
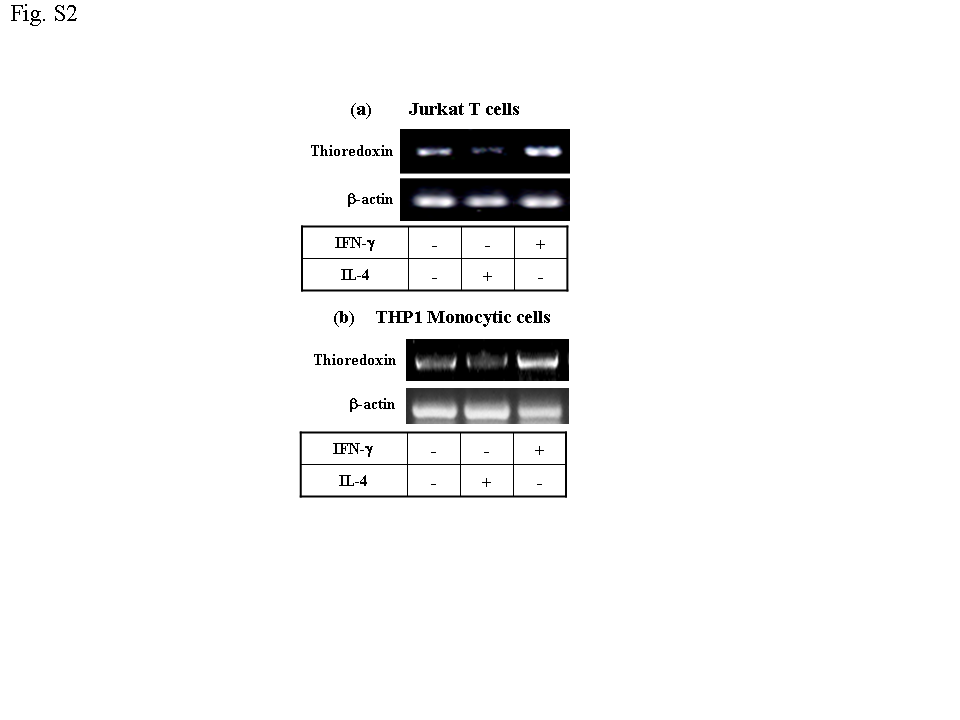
**

Supplement: Additional file 2 — RT-PCR analysis of thioredoxin gene expression induced by IFN-γ or IL-4 in immune cell lines. Jurkat T cells (2 × 10^6^) (Panel a) or THP1 monocytic cells (5 × 10^6^) (Panel b) were treated with media alone, IL-4, or IFN-γ as in Fig 1-A. The total RNA was then isolated and RT-PCR was performed using primers specific for the full-length thioredoxin cDNA. β-actin was used as an internal control. [file 1471-2172-9-64-S2.doc]
